# Neddylation: a novel modulator of the tumor microenvironment

**DOI:** 10.1186/s12943-019-0979-1

**Published:** 2019-04-03

**Authors:** Lisha Zhou, Yanyu Jiang, Qin Luo, Lihui Li, Lijun Jia

**Affiliations:** 1 Cancer Institute, Longhua Hospital, Shanghai University of Traditional Chinese Medicine, Shanghai, 200032 China; 2 Department of Biochemistry, Medical College, Taizhou University, Taizhou, 317000 Zhejiang China; 3 Department of Oncology, Cancer Institute, Fudan University Shanghai Cancer Center, Shanghai Medical College, Fudan University, Shanghai, 200032 China

**Keywords:** Neddylation, Tumor microenvironment, Tumor-derived factors, Cancer-associated fibroblasts, Cancer-associated endothelial cells, Immune cells

## Abstract

Neddylation, a post-translational modification that adds an ubiquitin-like protein NEDD8 to substrate proteins, modulates many important biological processes, including tumorigenesis. The process of protein neddylation is overactivated in multiple human cancers, providing a sound rationale for its targeting as an attractive anticancer therapeutic strategy, as evidence by the development of NEDD8-activating enzyme (NAE) inhibitor MLN4924 (also known as pevonedistat). Neddylation inhibition by MLN4924 exerts significantly anticancer effects mainly by triggering cell apoptosis, senescence and autophagy. Recently, intensive evidences reveal that inhibition of neddylation pathway, in addition to acting on tumor cells, also influences the functions of multiple important components of the tumor microenvironment (TME), including immune cells, cancer-associated fibroblasts (CAFs), cancer-associated endothelial cells (CAEs) and some factors, all of which are crucial for tumorigenesis. Here, we briefly summarize the latest progresses in this field to clarify the roles of neddylation in the TME, thus highlighting the overall anticancer efficacy of neddylaton inhibition.

## Introduction

**Fig. 1:**
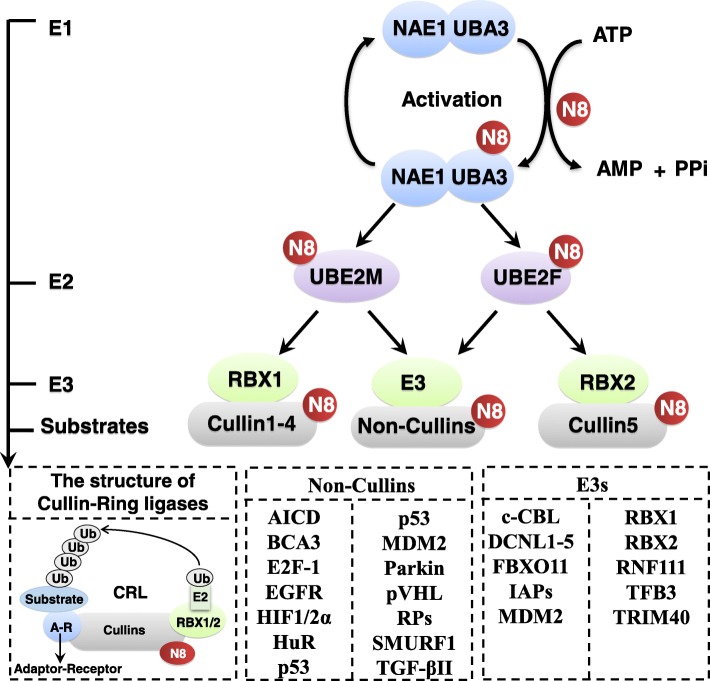
The process of protein modification by neddylation. Neddylation is a process of conjugating NEDD8, an ubiquitin-like molecule, to targeted protein substrates via enzymatic cascades involving NEDD8-activating enzyme E1, NEDD8-conjuagating enzyme E2 and substrate-specific NEDD8-E3 ligases. Shown are reported neddylation E1/E2s/E3s and substrates. The substrates were divided into cullins and non-cullins. N8: NEDD8

Neddylation is a reversible covalent conjugation of an ubiquitin-like molecule NEDD8 (neuronal precursor cell-expressed developmentally down-regulated protein 8) to a lysine residue of the substrate protein [1, 2]. Similar to ubiquitination, it is triggered by the successive enzymatic cascade of NEDD8-activating enzyme E1, NEDD8-conjuagating enzyme E2 and substrate-specific NEDD8-E3 ligases (Fig. 1) [3–5]. Briefly, the mature NEDD8 is first adenylated and activated by the E1 NEDD8-activating enzyme (NAE), a heterodimer consisting of NAE1 (also known as APPBP1) and UBA3 [6]. The NEDD8-loaded NAE is then transferred to one of two NEDD8-conjugating enzymes, UBE2M (also known as UBC12) or UBE2F through a trans-thiolation reaction [7, 8]. Ultimately, a substrate specific-E3 ligase, such as RBX1/2 or DCN1, transfers NEDD8 from E2 to a lysine residue in its target protein via covalent attachment (Fig. 1) [4, 5].

Overall, binding of NEDD8 molecules to target proteins can affect their stability, subcellular localization, conformation and function [4]. The best-characterized substrates of neddylation are the cullin subunits of Cullin-RING ligases (CRLs), which, as the largest family of multiunit E3 ubiquitin ligases, control degradation of about 20% of proteasome-regulated proteins, involving in many aspects of important biological processes [9–12]. Activation of CRLs requires the conjugation of NEDD8 to a key lysine residue at C-terminus of cullins to induce its conformational change, which dissociates the CRLs negative regulator CAND1 and facilitates the assembly of a functional CRLs, and subsequent substrate ubiquitination [13–17]. Given that overactivation of CRLs leads to cancer growth and development, targeting cullin neddylation appears to be an attractive approach for cancer treatment [18, 19]. Intensive studies have proven that NEDD8 and enzymes of neddylation pathway (e.g. NAE1/UBA3, UBE2M/UBE2F and NEDD8 E3 ligases) are often overexpressed in multiple human cancers, which is associated with disease progression and predicts poor patient survival [20–27]. Overactivated neddylation pathway leads to the elevated global neddylation of substrates, such as cullins, to promote consequent degradation of tumor suppressor (e.g. p21 and p27) and facilitate carcinogenesis and progression [4, 5]. Thus, validation of neddylation pathway as a target to inactivate CRLs is a promising anticancer strategy.

Recently, MLN4924 (also known as pevonedistat), a potent and highly selective small molecular inhibitor of NAE, was discovered to block protein neddylation through inactivating the first step of the neddylation cascade [12]. Structural analysis showed that MLN4924 forms a steady-state covalent adduct with NEDD8, which resembles the adenylate-NEDD8 adduct at the active site of NAE to block further enzymatic process [3, 28]. By doing so, MLN4924 effectively blocks cullin neddylation and inactivates CRLs, leading to accumulation of various CRLs substrates, thus triggering multiple cellular responses, including cell cycle arrest, apoptosis, senescence and autophagy in a cell-type dependent manner [12, 28–31]. For its potent antitumor activity and well-tolerated toxicity in preclinical studies [32–34], MLN4924 has been advanced into a series of phase I/II/III clinical trials for patients suffering from solid tumors and hematological malignancies. To date, 30 clinical trials have been enrolled in clinicaltrials.gov website (https://www.clinicaltrials.gov/), and five completed phase I clinical trials demonstrated that MLN4924 is safe and feasible, as best evidenced by the partial response (PR) completed responses (CR) and prolonged stable disease (SD) (summarized in Table 1) [35–40]. Given those promising clinical effects, several phase II clinical trials are currently recruiting patients. Notably, another one phase III trial has been launched in combination of MLN4924 with azacitidine in patients with acute myelogenous leukemia (AML), myelodysplastic syndrome (MS) and chronic myelomonocytic leukemia (CMML).

**Table 1:**
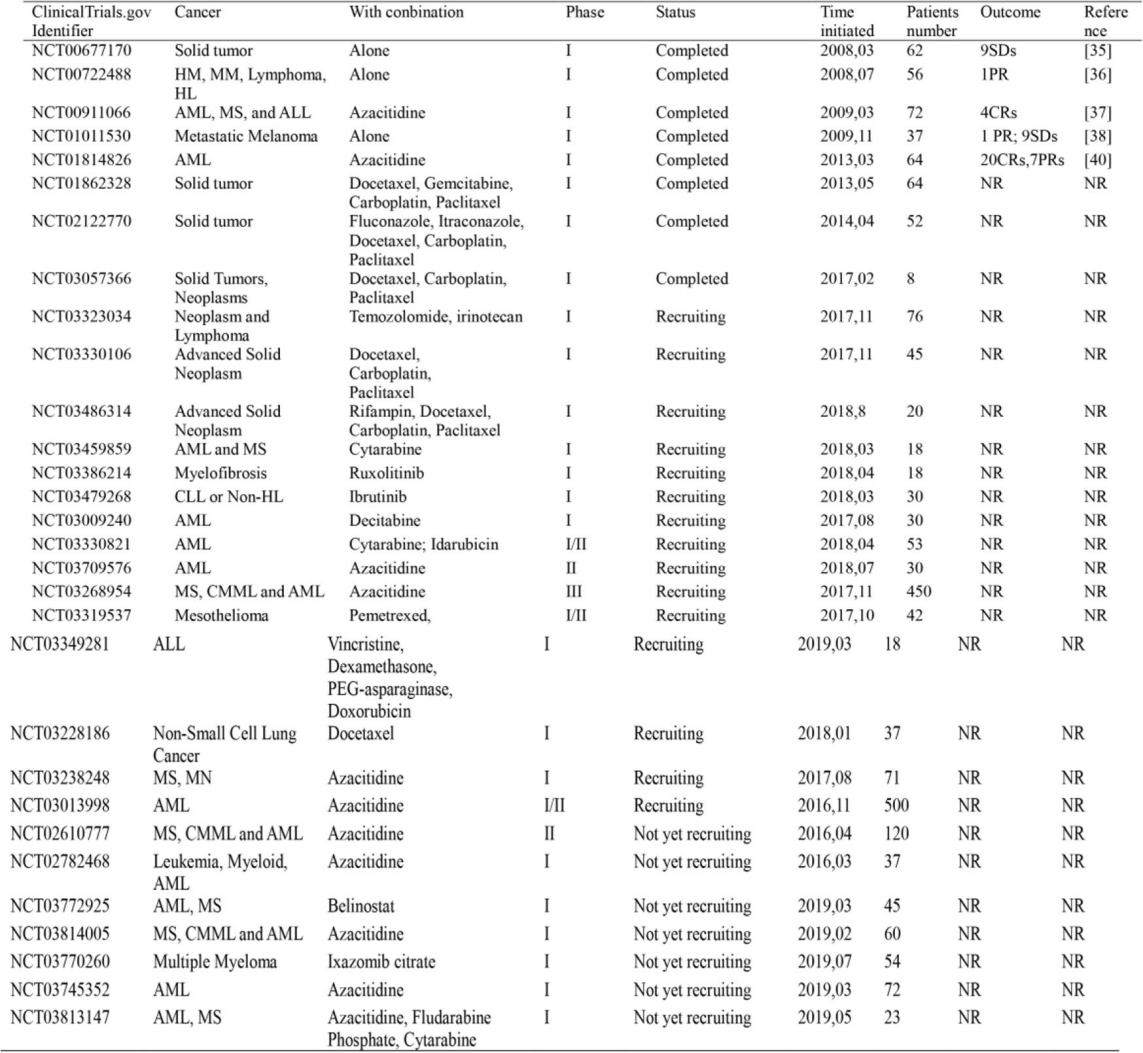
Clinical Trials of MLN4924

*CR* Complete response, *PR* Partial Response, *SD* Stable Disease, *NR* Not Reported, *AML* Acute Myelogenous Leukemia, *MS* Myelodysplastic Syndromes, *ALL* Acute Lymphoblastic Leukemia, *CMML* Chronic Myelomonocytic Leukemia, *CLL* Chronic Lymphocytic Leukemia, *MN* Myeloproliferative Neoplasm, *HL* Hodgkin lymphoma, *HM* Hematologic Malignancies, *MM* Multiple Myeloma

Intriguingly, increasing studies highlight the significant role of neddylation in the regulation of the tumor microenvironment (TME) [5], which in addition to tumor cells, comprises immune cells, cancer-associated fibroblasts (CAFs), cancer-associated endothelial cells (CAEs) and some factors [41, 42]. These components of TME play a crucial role in facilitating tumor progression, and targeting these cells might determine the fate of the tumor [41, 42]. In this review, we summarize the roles of neddylation pathway in regulating the functions of TME, to deepen our understanding on the significance of neddylation in regulation of tumor progression, and further validate neddylation as a promising anticancer target.

### Neddylation pathway plays a crucial role in the modulation of TME

#### Neddylation as a modulator of tumor-derived factors

**Fig. 2:**
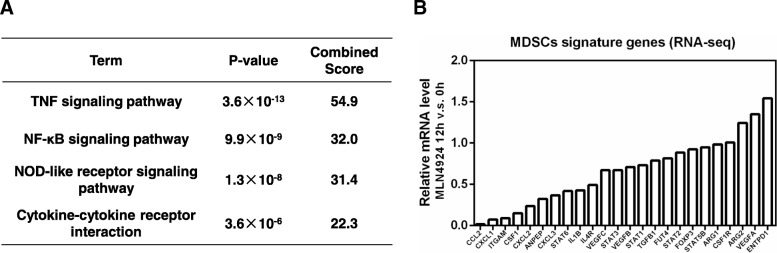
Neddylation acts as a modulator of tumor-derived factors. **a** KEGG pathway enrichment analysis of down-regulated genes induced by MLN4924 in lung cancer cells. **b** Most of the 22 MDSCs-related genes were down-regulated with MLN4924 treatment. H1299 lung cancer cells treated with 1 μM MLN4924 for 12 h, were used for gene expression profiling

The tumor microenvironment is generated by the tumor and dominated by tumor-induced interactions [42]. For example, tumor-infiltrated immune cells are enriched in myeloid-derived suppressor cells (MDSCs), which contribute to a immunosuppressive microenvironment; while, anti-tumor functions are down-regulated, largely in response to tumor-derived signals [42]. We first determined the differentially expressed genes associated with neddylation inhibition by MLN4924 in lung cancer cells in vitro. Intriguingly, pathway enrichment analysis with KEGG showed that, many inflammatory/immune-related pathways were significantly enriched with the down-regulated differentially expressed genes (Fig. 2a). The top two pathways were TNF signaling pathway and NF-κB signaling pathway (*P* < 0.001), both of which play critical roles in the production of immune-associated factors [43] (Fig. 2a). Among them, the expressions of CCL2 and CXCL1, two chemokines important for MDSCs recruitment in tumors [44], were decreased obviously (Fig. 2b). Then, we generated a list of 22 MDSCs-related genes curated from literature analysis to link the neddylation pathway and MDSCs activation in lung cancer cells [45]. Markedly, most of the 22 MDSCs-related genes are significantly down-regulated with MLN4924 treatment (Fig. 2b). Next, we established a lung metastasis mice model by intravenously injecting the aggressive murine Lewis lung carcinoma (LLC) cells. Significantly decreased proportion of the Gr-1^+^CD11b^+^ MDSCs was found in MLN4924-treated or NEDD8-knockout tumor-bearing lungs compared with these from control group (unpublished data), supporting the notion that neddylation pathway plays a crucial role in the modulation of MDSCs infiltration in tumor sites. Together, these results suggest that the overactivation of neddylation pathway in tumor cells might regulate tumor-derived signals to ameliorate a tumor-promoting microenvironment.

#### Neddylation as a modulator of fibroblasts

**Fig. 3:**
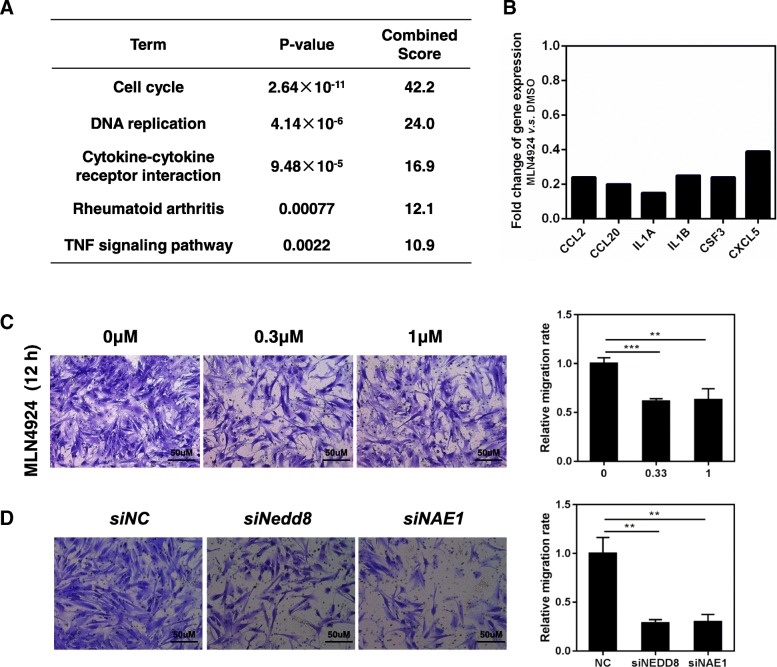
Neddylation acts as a modulator of cancer-associated fibroblasts (CAFs). **a** KEGG pathway enrichment analysis of down-regulated genes induced by MLN4924 in CAFs. CAFs were isolated from hepatocellular carcinoma (HCC) tissues, and treated with 1 μM MLN4924 for 12 h. **b** The expression of several inflammatory cytokines was reduced upon MLN4924 treatment. **c**-**d** Neddylation inhibition, either by MLN4924 treatment or siRNA-mediated depletion of NEDD8 or NAE1, suppressed CAFs migration. Conditioned medium (CM) collected from supernatants of HCC cells was used for chemotaxis assay. 5 × 10^4^ isolated CAFs were placed into the upper chamber and treated with MLN4924 for 12 h at 37 °C. Cells that migrated were fixed and stained, followed by counting the cell number under a Leica microscope to measure. NC: Negative control. Scale bar for× 200 images, 50 μm

Cancer-associated fibroblasts (CAFs), a major stromal component, play important roles in regulating angiogenesis and metastasis of tumor cells by releasing growth factors, inflammatory cytokines and chemokines [46, 47]. Therefore, targeting CAFs may serve as an effective approach for cancer treatment [48]. Recently, our group found that neddylation pathway is a key regulator of CAFs activation. CAFs were isolated from hepatocellular carcinoma (HCC) tissues [49], and treated with MLN4924 to determine the differentially expressed genes. RNA sequencing analysis firstly revealed that the levels of 406 genes (165 increased, 241 decreased) were significantly altered following treatment with MLN4924 compared to control CAFs. Interestingly, we detected that significant down-regulation of genes were involved in cell cycle and DNA replication pathways (Fig. 3a), indicating that the proliferation and activation of CAFs might be inhibited by MLN4924 treatment.

CAFs are well-characterized by overactivation of genes related to inflammation and chemotaxis of immune cells [50, 51]. Among them, CCL2 is highly expressed compared with normal fibroblasts and contributes to CAF-mediated tumor-promoting inflammation [52]. Our data showed that the expression of several inflammatory cytokines, including CCL2, were reduced upon MLN4924 treatment (Fig. 3b). To further determine whether the activation of CAFs was inhibited by MLN4924, we detected the migration rate of CAFs, which is also used to evaluate its tumor-promoting activation. As shown, either by MLN4924 treatment or siRNA-mediated depletion of NEDD8 or NAE1, obviously suppressed the CAFs migration (Fig. 3c and d). Collectively, our findings point out the important role of neddylation pathway for CAFs activation partially through influencing the cell proliferation, migration and tumor-promoting cytokines secretion. Mechanistic understanding of these inhibitory effects of neddylation inhibition on CAFs awaits further investigation.

#### Neddylation as a modulator of endothelial cells

Endothelial cells are important components of TME, contributing considerably to angiogenesis and regulation of tumor metastasis [53, 54]. Recent studies showed that MLN4924 treatment significantly decreases the levels of total NEDD8-conjugated proteins and Cullins neddylation to suppress the formation of capillary-like tube networks, transwell migration and migrated distance of human umbilical vein endothelial cells (HUVECs) as well as mouse endothelial cells (MS-1) in a dose-dependent manner [21, 55]. Consistently, genetic deletion of RBX2, a neddylation E3 ligase, recapitulates the anti-angiogenic effect of MLN4924 in HUVECs [56]. Moreover, several classical angiogenic assays (e.g. aortic ring, CAM and matrigel-plug) were also used to support the suppressive effect of MLN4924 on angiogenesis in vitro [55, 56]. Much more importantly, MLN4924 suppresses in vivo tumor angiogenesis, progression and metastasis in orthotopic models of pancreatic cancer [55, 56]. These findings show that inhibition of neddylation via pharmacological or genetic approaches suppresses endothelial cells activation and tumor angiogenesis.

**Fig. 4:**
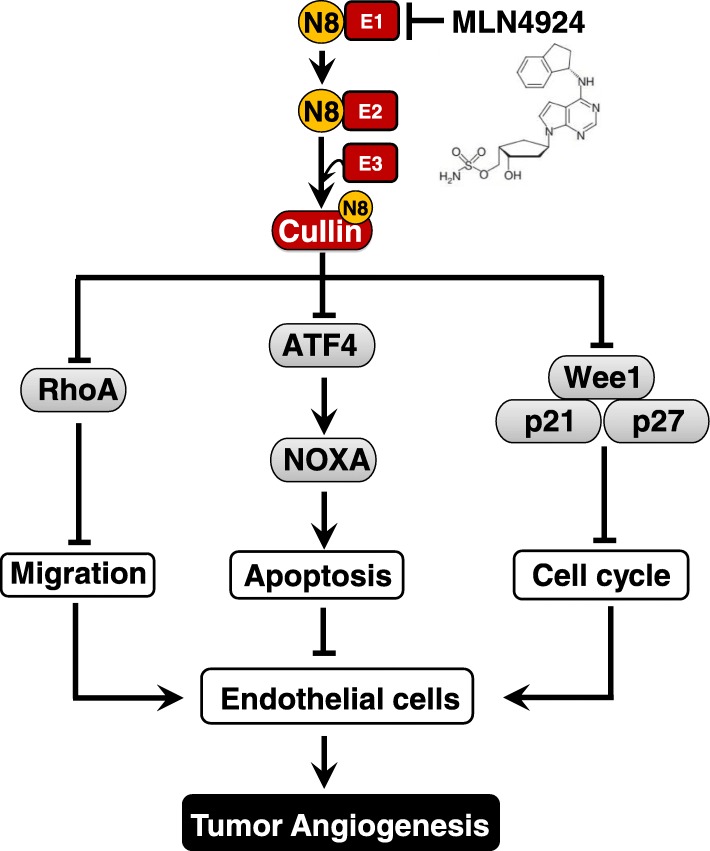
Inhibition of neddylation pathway impairs migration, proliferation and survival of endothelial cells by accumulation of CRL substrates

Mechanistically, the activity of MLN4924 against endothelial cells activation is largely mediated by inactivating CRLs and subsequently accumulating different sets of CRLs substrates [55]. At early stages post treatment, when cell viability is not obviously disturbed, the suppressive effect of MLN4924 on endothelial cells is attributed to the accumulation of CRLs substrate RhoA, which inhibits cell migration and capillary tube formation [55, 57, 58] (Fig. 4). With prolonged exposure time, MLN4924 induces the accumulation of cell cycle-related CRLs substrates (e.g. p21, p27 and WEE1), pro-apoptotic protein (e.g. NOXA, which was transactivated by CRLs substrate ATF4), leading to DNA damage response, cell cycle arrest and apoptosis of endothelial cells [55, 56, 59] (Fig. 4).

Collectively, these data highlight the importance of neddylation pathway in endothelial cells, and support the notion for the development of neddylation inhibitors (e.g. MLN4924) as novel class of anti-angiogenic and anti-tumor agents [55].

#### Neddylation as a modulator of infiltrated immune cells

**Fig. 5:**
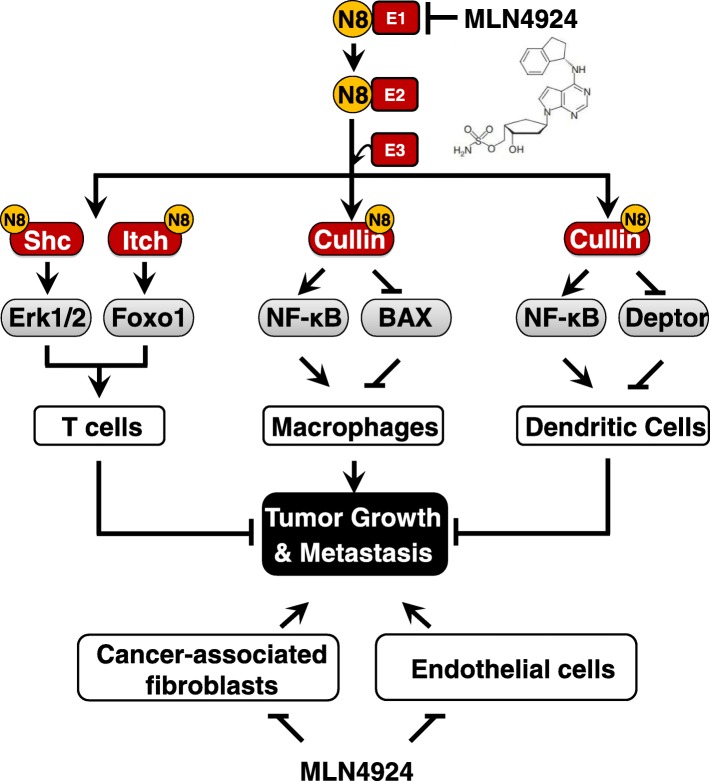
Neddylation pathway plays a crucial role in the modulation of TME. 1) Neddylation inhibition suppresses the activation of tumor-associated fibroblasts (CAFs) and tumor-associated endothelial cells (CAEs). 2) Neddylation inhibition suppresses immune cells, including T cells, dendritic cells and macrophages

Different types of infiltrated immune cells are involved in the TME and play critical roles in all stages of tumor development from initiation, promotion, and progression to metastasis [60]. Thus, targeting these immune cells is likely to be a promising anti-cancer strategy [60, 61]. Recently, a number of studies have implicated a potential role of neddylation modification in the regulation of functions of several immune cells, including macrophages, T-cells and dendritic cells (DCs) (Fig. 5).

### Neddylation as a modulator of macrophages

Macrophages present in tumors are known as tumor-associated macrophages (TAMs). TAMs are key components of the TME, altering the tumor microenvironment to accelerate tumor progression and metastasis through the induction of growth factors, angiogenic regulators and inflammatory mediators [62, 63]. Recent studies showed that inactivation of neddylation inhibits inflammatory responses of macrophages. It was reported that blocking neddylation, either pharmacologically (e.g. MLN4924) or genetically (e.g. siRNA), represses lipopolysaccharides (LPS)-induced production of proinflammatory cytokines (e.g. TNF-α and IL-6) in macrophages, through inhibiting Cullin1-mediated IκB degradation to block NF-κB translocation and transcriptional activation [64, 65]. Apart from promoting the functions of macrophages, neddylation pathway is also essential for their proliferation and survival, through facilitating cell-cycle progression and preventing apoptosis [65].

Similarly, manipulation of RBX2 was found to regulate macrophage survival/death and immune response when challenged by pathogen-associated molecular patterns (PAMPs) [66]. Specifically, RBX2 knockdown in macrophage causes the accumulation of pro-apoptotic proteins (e.g. BAX) to induce apoptosis [66]. Moreover, RBX2 overexpression triggers upregulation of pro-tumorigenic cytokines (IL-1β, IL-6 and TNFα), and downregulation of anti-tumorigenic cytokine (IL-12) and anti-inflammatory cytokine (IL-10) [66].

Given that macrophages are critical for tumor progression and dysregulation of neddylation pathway do affect its functions, targeting neddylation pathway in macrophages could be a new approach for cancer therapy. Currently, the effects and underlying mechanisms of targeting neddylation pathway in macrophages for the treatment of cancers are under investigation in our lab.

### Neddylation as a modulator of T cells

T cells-mediated immunity plays a critical role in immune responses against cancer [67, 68]. T-cell activation is initiated by the engagement of T-cell antigen receptor (TCR) and co-stimulatory molecules, which ultimately leads to proliferation, cytokine production and differentiation into different types of T helper (Th) cells [67]. Emerging evidences show that neddylation pathway is an important modulator of T cells activation. Blockade of neddylation pathway either by MLN4924 treatment or siRNA-mediated depletion of UBE2M induces CD4^+^ T cells G0/G1 phase arrest, leading to much slower division than control T cells [69]. Moreover, neddylation inhibition leads to impaired antigen-driven cytokine production (e.g. IFN-γ, IL-2 and IL-4), which is required for efficient Th1 and Th2 differentiation [70], demonstrating a potent positive function of neddylation pathway in T-cell activation [69, 71]. Consistently, deletion of RBX2 significantly decreased T-cell activation and T-effector cytokine release upon in vitro allogeneic stimulation [72].

Mechanistically, the activation of the extracellular regulated protein kinases (Erk)1/2, an essential regulator of T-cell biology [73], is profoundly impaired in the neddylation inhibition CD4^+^ T cells [69]. Interestingly, neddylation pathway seems to directly regulate Shc to facilitate the formation of a ZAP70-Shc-Grb2 signaling complex and affect downstream Erk activation [69, 74]. Subsequently, Cheng et al. reported that neddylation pathway is required for supporting various aspects of CD4^+^ T-cell functions, through B cell lymphoma-2 (Bcl-2)-mediated suppression of the mitochondria-dependent apoptosis [71]. Moreover, neddylation contributes to follicular Th cells differentiation, probably via augmenting the activity of ubiquitin ligase activity Itch by a mono-neddylation process and subsequent proteasomal degradation of FOXO1, a transcription factor implicated in multiple aspects of T-cell functions [71, 75]. Collectively, these findings indicate that inhibition of neddylation pathway acts as crucial modulators of T cells activation and anti-tumor immune response.

### Neddylation as a modulator of dendritic cells

Dendritic cells (DCs) play central roles in the induction of anti-tumor immunity, providing critical signals that drive the expression of cytokines and co-stimulatory molecules to strengthen ability in T cells activation [76–78]. Notably, neddylation pathway in DCs is associated with the activity of DCs and its immune regulation. Researchers found that MLN4924 remarkably suppresses the production of cytokines TNF-α and IL-6, which represent acute inflammatory response [79–81]. The inhibitory effect is further supported by the siRNA knockdown of RBX2 [80]. Moreover, the secretion of IL-12p70, a key cytokine produced by DCs for Th1 differentiation [82], and the expression of co-stimulatory molecules, are significantly suppressed with MLN4924 treatment, suggesting the restricted capacity in T-cell activation and immune responses [79]. In addition, MLN4924 treatment or NEDD8 knockdown could trigger the apoptosis or necroptosis of DCs in the caspase-dependent manner, resulting in the reduction of functional DCs [79, 83].

In terms of mechanism, the accumulation of Deptor, an inhibitory protein of mTOR, is involved in MLN4924-induced inhibitory effects on DCs [79]. In detail, MLN4924 inhibits Cullin1 neddylation and weakens its ability in the degradation of Deptor [79, 84, 85], thus leading to mTOR inactivation and consequent DCs functional suppression [79, 86, 87]. Meanwhile, Mathewson et al. showed that NF-κB signaling is also involved in altering cytokine production in DCs, when subjected to MLN4924 [80]. MLN4924 suppresses the release of TNF-α and IL-6 through the inactivation of CRL-1, thus causing IκBα accumulation and subsequent prevention of NF-κB activation [80, 88]. In addition, some of other neddylation substrates, such as Cullin-2 and HIF-1α, could also influence DCs activation and maturation [89, 90]. On the whole, neddylation inhibition might suppress DCs functions via modulating multiple signaling pathway in a neddylation-dependent manner according to diverse NEDD8-conjugating proteins.

## Conclusion

Recent and ongoing investigations highlight a pivotal role of neddylation pathway in tumor biology and immune cell development. Neddylation pathway can effect tumor progression by regulating multiple cellular responses of tumor cells (e.g. apoptosis and senescence) or modulating the functions of stromal cells in the TME (e.g. angiogenesis and immune responses) (Fig. 5), supporting the notion that inhibition of this pathway is a novel and promising anti-tumor therapeutic strategy [5]. A good example is the development of MLN4924, which has been currently investigated in many phase I/II/III clinical trials for its potent antitumor activity and well-tolerated toxicity [39]. Intensive studies are directed to the following aspects for the advancement of the neddylation-TME field.

First, the roles of neddylation in tumor and stromal cells have been thoroughly reviewed here and elsewhere [4, 5], while the neddylation-mediated crosstalk between tumor cells and stromal cells in TME is still not reported. The TME is created and dominated by tumor cells through various types of crosstalk [42]. Our group found that neddylation inhibition by MLN4924 in lung cancer cells suppresses the expression of several tumor-derived inflammatory factors, which are crucial for generation of the tumor-promoting immune microenvironment. Thus, regulatory mechanisms of neddylation pathway on tumor-derived signals and subsequent functions await further investigation.

Second, as mentioned above, several stromal cells (e.g. CAFs, CAEs and macrophages), which exert the tumor-progressive effects, require the neddylation pathway to maintain its activation; while the functions of T cells and dendritic cells, which contribute to anti-tumor immunity, are also impaired by neddylation inhibition. It is, therefore, anticipated to suffer from toxicity of anti-tumor immune cells in clinical trials. In other words, it is important to assess the effect of neddylation inhibition by MLN4924 on the proportion of immune cells in vivo. So far, no relevant research has been reported yet. Additionally, to avoid MLN4924 being taken up by normal cells or anti-tumor immune cells, development of proper delivery vehicle as carriers of MLN4924 (e.g. tumor-specific nanoparticle) could be the future direction.

Third, whether and how does the elevated neddylation pathway in cancer cells contribute to create a tumor-promoting microenvironment? How significant does the disturbance of tumor microenvironment by neddylation inactivation contribute to the overall anticancer efficacy of neddylation inhibitor (e.g. MLN4924)? Once confirmed, identification of useful biomarkers of different TME components respond to neddylation inhibition will certainly help to evaluate the therapeutic efficacy of neddylation inhibitors.

In summary, the data we have summarized here clearly indicate a critical role of neddylation pathway in the TME and lay a further foundation for neddylation-based therapies in cancer treatment.

## Abbreviations

BAX: BCL2-associated X
BCL2: B cell lymphoma-2
CAEs: Cancer-associated endothelial cells
CAFs: Cancer-associated fibroblasts
CAM: Chorioallantoic membrane
CAND1: Cullin associated and neddylation dissociated 1
CCL2: Chemokine (C-C motif) ligand 2
CRLs: Cullin-RING ligases
DCN1: NEDD8 ligase DCN1
DCs: Dendritic cells
Deptor: Dep domain containing mTOR interacting protein
Erk1/2: Extracellular regulated protein kinases 1/2
FOXO1: Forkhead box O1
HIF-1α: Hypoxia inducible factor 1 subunit alpha
HUVECs: Human umbilical vein endothelial cells
IFN-γ: Interferon-γ
IL-10: Interleukin 10
IL-12: Interleukin 12
IL-1B: Interleukin 1 beta
IL-2: Interleukin 2
IL-4: Interleukin 4
IL-6: Interleukin 6
Itch: Itchy E3 ubiquitin protein ligase
IκB: Inhibitor of nuclear factor kappa B
MDSCs: Myeloid-derived suppressor cells
MS-1: Mouse endothelial cells
mTOR: Mechanistic target of rapamycin kinase
NAE1: NEDD8 activating enzyme E1 subunit 1
NEDD8: Neuronal precursor cell-expressed developmentally down-regulated protein 8
NF-κB: Nuclear factor kappa B
PAMPs: Pathogen-associated molecular patterns
RBX1/2: RING-box protein 1/2
Shc: Shc-adaptor protein 1
TAMs: Tumor-associated macrophages
Th: T helper
TME: Tumor microenvironment
TNFα: Tumor necrosis factor α
UBA3: Ubiquitin like modifier activating enzyme 3
UBE2F: Ubiquitin conjugating enzyme E2 F
UBE2M: Ubiquitin conjugating enzyme E2 M
